# A Novel Method of Predicting Protein Disordered Regions Based on Sequence Features

**DOI:** 10.1155/2013/414327

**Published:** 2013-04-22

**Authors:** Tong-Hui Zhao, Min Jiang, Tao Huang, Bi-Qing Li, Ning Zhang, Hai-Peng Li, Yu-Dong Cai

**Affiliations:** ^1^Institute of Systems Biology, Shanghai University, Shanghai 200444, China; ^2^Department of Mathematics, College of Science, Shanghai University, Shanghai 200444, China; ^3^State Key Laboratory of Medical Genomics, Institute of Health Sciences, Shanghai Jiaotong University School of Medicine and Shanghai Institutes for Biological Sciences, Chinese Academy of Sciences, Shanghai 200025, China; ^4^Department of Genetics and Genomic Sciences, Mount Sinai School of Medicine, New York, NY 10029, USA; ^5^Key Laboratory of Systems Biology, Shanghai Institutes for Biological Sciences, Chinese Academy of Sciences, Shanghai 200031, China; ^6^Department of Biomedical Engineering, Tianjin University, Tianjin Key Lab of BME Measurement, Tianjin 300072, China; ^7^CAS-MPG Partner Institute for Computational Biology, Shanghai Institutes for Biological Sciences, Chinese Academy of Sciences, Shanghai 200031, China

## Abstract

With a large number of disordered proteins and their important functions discovered, it is highly desired to develop effective methods to computationally predict protein disordered regions. In this study, based on Random Forest (RF), Maximum Relevancy Minimum Redundancy (mRMR), and Incremental Feature Selection (IFS), we developed a new method to predict disordered regions in proteins. The mRMR criterion was used to rank the importance of all candidate features. Finally, top 128 features were selected from the ranked feature list to build the optimal model, including 92 Position Specific Scoring Matrix (PSSM) conservation score features and 36 secondary structure features. As a result, Matthews correlation coefficient (MCC) of 0.3895 was achieved on the training set by 10-fold cross-validation. On the basis of predicting results for each query sequence by using the method, we used the scanning and modification strategy to improve the performance. The accuracy (ACC) and MCC were increased by 4% and almost 0.2%, respectively, compared with other three popular predictors: DISOPRED, DISOclust, and OnD-CRF. The selected features may shed some light on the understanding of the formation mechanism of disordered structures, providing guidelines for experimental validation.

## 1. Introduction

The protein structure-function paradigm has been believed as a dogma in the 20th century. However, the discovery of intrinsically disordered proteins, which have regions devoid of stable secondary structures or have a large number of conformations [1], challenges the traditional view and calls for reassessment of the paradigm. 

Eukaryotic proteins apparently have more intrinsic disordered regions than those of bacteria or archaea [2], suggesting also more important functions such as being involved in signaling and regulation of gene expression [3]. Lack of intrinsic structures could render protein additional functions, including binding to different targets [4], transcriptional regulation, translational regulation, and cellular signal transduction regulation [5].

Although there is a growing amount of disordered proteins discovered or shown to have disordered regions under physiological conditions [6], most of them were poorly detected by experimental approaches [2, 4, 5, 7–9]. Firstly, such experimental methods are often time consuming and expensive. Furthermore, it is believed, in X-ray crystallography, that regions missing electron density were related to disorder in many protein structures [6]. However, without additional experiments, it is not sure whether a low electron-density region is intrinsically disordered or is a wobbly domain, or just the result of technical difficulties [2]. NMR spectroscopy, one of the most readily suited techniques for detecting disordered proteins in solution, could also underrepresent a native molten globular domain, which is one of the types of disordered regions [2].

Generally speaking, intrinsically disordered proteins have a biased amino acid composition. Weathers and colleagues reported that amino acid composition was sufficient to be used to accurately recognize disorder [10]. Several algorithms for predicting intrinsically disordered proteins have been developed, such as DISOPRED [11], DISOclust [12], and OnD-CRF [13]. DISOPRED is a web service, which is trained on high resolution X-ray crystal structures and identifies disorder when the electron density map of a residue misses coordinates. It is initially generated sequence profile by a PSI BLAST [14] searching. After being trained using a support vector algorithm, the classifier can output a probability estimate. However, a limitation of this algorithm is that coordinates missing may be caused by the artifact of the crystallization process rather than disorder. DISOclust, based on analysis of three-dimensional structure models, identifies disorder when residues change or are consistently missing. OnD-CRF is a method for predicting the transition between structured and disordered regions. The approach uses conditional random fields relying on features derived from amino acid sequences and secondary structure prediction results. 

In the present study, we developed a new strategy for analyzing and predicting protein disordered regions by means of Random Forest (RF), Maximum Relevancy Minimum Redundancy (mRMR), Incremental Feature Selection (IFS), and a scanning and modification strategy. Optimal feature set was selected from candidate features, containing Position Specific Scoring Matrix (PSSM) conservation score features and secondary structure features. Our method outperformed other three existing disorder predictors achieving the highest ACC and MCC values. 

## 2. Materials and Methods

### 2.1. Benchmark Dataset

In this study, disordered proteins were downloaded from the Database of Protein Disorder (DisProt) (version 4.9) [8], which is constructed based on literature description, providing structured and functional information for intrinsically disordered proteins. Ordered proteins were collected from DisProt database and PDB-Select-25 (the October 2008 version) [15]. PDB-Select-25 is a representative subset of the Protein Data Bank (PDB), containing protein families less than 25% sequence identity [16]. Data was preprocessed according to the following criteria. (i) Only disordered protein chains having more than 50 residues and only proteins with low resolution (≥2 Å) were retained. (ii) Only chains having no missing backbones or side chain coordinates were retained. Finally, 960 protein chains containing 293,780 residues were obtained, in which 55,637 residues were in disordered regions. All protein chains were divided randomly into training set and test set.

A 21-residue sliding window approach was employed along each of the protein sequence, containing the center ordered or disordered residue and 10 residues upstream and downstream of the center residue. Since the dataset used in this study was an unbalanced dataset with much more ordered samples than disordered ones, for the training set, we randomly selected the equal number of ordered samples to match the disordered ones. Finally, 43,903 ordered samples and 43,903 disordered samples from 753 proteins were obtained in the training set, which can be found in Online Supporting Information S1 available online at http://dx.doi.org/10.1155/2013/414327. The test set contained 54,582 ordered samples and 11,734 disordered samples from 192 proteins, which were given in Online Supporting Information S2.

### 2.2. Feature Extraction

#### 2.2.1. Feature of PSSM Conservation Scores

Evolutionary conservation is considered important in biological sequence analysis. A more conserved residue within a protein sequence indicates that it is under stronger selective pressure and hence more important for the protein function. Mutations on such residues may cause significant changes of the protein. In view of this, we used conservation scores to encode peptides. 

Herein, the Position Specific Iterative BLAST (PSI BLAST) was employed to measure the conservation status for a specific residue. For each residue, a 20-dimensional vector was calculated to denote the conservation probabilities of mutations to 20 basic amino acids. For a given protein sequence, a Position Specific Scoring Matrix (PSSM [17]) was obtained, which was constructed by all vectors of all residues in the sequence. 

#### 2.2.2. Feature of Secondary Structures

Intrinsically disordered proteins are devoid of well-defined tertiary structures under physiological conditions; however, generally speaking, they often display signs of local secondary structures [18, 19]. After statistical analysis of complex of 24 intrinsically disordered proteins, Fuxreiter et al. [20] found that some regions in disordered proteins had strong preference for helical structures. Therefore, in this study, each amino acid was encoded as three types of secondary structures: helix, strand, or coil, as predicted by SSPro [21]. Helix, strand, and coil are the three major kinds of protein secondary structures. Helix is the protein region with spiral conformation. Strand is a protein structural unit of twisted, pleated sheet. The coil region is the region that does not belong to helix or strand. SSPro predicts the protein secondary structures based on PSI-BLAST profiles with an ensemble neural network model [21].

Thus, each 21-residue peptide was encoded into a vector containing (20 + 3) × 21 = 483 features. The features are named with following rules: first, the amino acid position (“AA” with position), then, feature types (“PSSM” and “SS”), and last, detail information. For PSSM features, it is the amino acid type. For secondary structure (SS) features, it is the secondary structure code. In secondary structure code, H, E, and C strand for helix, strand, and coil, respectively.

### 2.3. Maximum Relevancy Minimum Redundancy (mRMR)

A classification model containing more features may not have more discriminating power. Additional features may have detrimental effects on the classification such as slowing down the learning process and causing overfitting the training data. It is believed that feature selection is an effective way of reducing the dimension of the feature space to improve the prediction performance.

The Maximum Relevancy Minimum Redundancy (mRMR) method was used in this study to select an optimal feature subset. The mRMR was originally developed by Peng et al. [22] to deal with the microarray data processing. If a feature had better tradeoff between maximum relevance to the target and minimum redundancy among other features, it was deemed as a better feature and would be ranked first (with a smaller index) in the final ordered list. The algorithm is described briefly below.

To determine the relevance properties of the feature space, the mutual information (MI), denoted as *I*, is defined as

(1) $$\begin{array}{c} I\left(x,y\right)=\iint P\left(x,y\right)\log\frac{P\left(x,y\right)}{P\left(x\right)P\left(y\right)}dx\;dy, \end{array}$$

where *x* and *y* are two random variables. *P*(*x*, y) is the joint probabilistic density function of *x* and *y*. *P*(*x*) and *P*(*y*) are the margin probabilistic density functions of *x* and *y*, respectively. To calculate MI, the joint probabilistic density function *P*(*x*, *y*) and the margin probabilistic density functions *P*(*x*) and *P*(*y*) should be given in advance.

Suppose *G* denotes the entire feature space; we aim to find a subset *S* of the features to satisfy both maximum relevance and minimum redundancy.

Based on MI, the following mRMR function is constructed:

(2) $$\begin{array}{c} \underset{f_{j}\in \Omega_{t}}{\max}\left[I\left(f_{j},c\right)-\frac{1}{m}\sum_{f_{i}\in \Omega_{s}}I\left(f_{j,}f_{i}\right)\right]\;\left(j=1,2,\ldots ,n\right), \end{array}$$

where *Ω*_*s*_ is the already selected feature set and *Ω*_*t*_ is the to-be-selected feature set, and *m* and *n* are the sizes of these two feature sets, respectively. The higher the ordered rank is, the more important the feature is.

A parameter is introduced here to deal with the continuous variables. In our study, *t* was assigned to be 1. Finally, an ordered feature list was obtained in which better features had smaller indexes.

The mRMR software could be obtained from http://penglab.janelia.org/proj/mRMR/.

### 2.4. The Random Forest (RF) Method

The Random Forest (RF) algorithm, firstly introduced by Svetnik [23] in 2003, is a combining ensemble tree-structured classifier. The individual decision tree in the forest depends on a random vector and has independent identically distribution. The Random Forest has been widely used in various fields such as economics and medical and text categorization. It has been also successfully employed in biological prediction problems [24–26] and even can efficiently handle large-scale dataset.

 In our research, we use the Random Forest (RF) algorithm to construct a prediction model to predict whether an amino acid is in disordered region or not. The method is briefly introduced as follows.

 Firstly, 10 decision trees are grown according to the following criteria.

- Suppose the number of cases in the training data is *M*; sample *M* cases randomly with replacement from the original data to keep the size of the original data not changing.
- When dealing with each note, *n* predictors are selected randomly in terms of *N* features (where *n* ≪ *N*). The split on the *n* predictors is also implemented to split the corresponding note. The m value is set to constant.
- Each tree is grown as large as possible and unnecessarily pruned. Then each tree gives the queried input a classification. Finally, the forest will choose the one that has the most votes among the trees.

In this study, the Random Forest classifier in Weka was employed with default parameters. The WEKA program is available at http://www.cs.waikato.ac.nz/ml/weka/downloading.html.

### 2.5. The Cross-Validation Method

In the literature, cross-validation methods are used to evaluate the stability of a predictor. The independent dataset test, subsampling (*k*-fold cross-validation), and jackknife analysis are the three methods generally used [27]. For a given benchmark dataset, the jackknife test generates a unique outcome and is deemed as the most objective one compared to other two methods, as elucidated in [28, 29] and demonstrated by [30, equations (28)–(32)] in. However, to reduce the computational time, in this study, 10-fold cross-validation test was used instead of jackknife test. During the 10-fold cross-validation, the whole dataset is divided into 10 equal parts. Each part is in turn used as test set and the remaining 9 parts as training set. We introduced prediction accuracy (ACC), specificity (SP), sensitivity (SN), and Matthews correlation coefficient (MCC) to evaluate the performance of the predictor, which are calculated as follows:

(3) ACC=TP+TNTP+TN+FP+FN,SN=TPTP+FN,SP=TNTN+FP,MCC =TP×TN−FP×FN(TP+FN)×(TN+FP)×(TP+FP)×(TN+FN),

where TP, TN, FP, and FN stand for the number of true positive, true negative, and false positive, false negative samples, respectively.

### 2.6. Incremental Feature Selection (IFS)

The incremental feature selection (IFS) [31–33] procedure was used to find an optimal subset from the mRMR feature list generated above. Suppose the total number of the features is *N*; we can obtain *N* feature subsets which are initiated from a subset containing one feature and generated by adding them one by one from the mRMR feature list.

 The *i*th subset is denoted by

(4) $$\begin{array}{c} S_{i}=\left\{f_{1},f_{2},\ldots ,f_{i}\right\}\;\left(1\leq i\leq N\right). \end{array}$$

Based on the *N* feature subsets, *N* Random Forest predictors were constructed with 10-fold cross-validation evaluating its performance. Then the IFS curve of MCC to the feature subset index *i* was plotted, in which the peak point was noted as *h*. Finally an optimal feature subset was obtained with which the corresponding predictor yields the best MCC.

## 3. Results and Discussion

### 3.1. Feature Reduction

We calculated the Cramer's *V* coefficient [34, 35] between features and targets. The Cramer's *V* coefficient is a statistical measurement derived from the Pearson chi-square test [36]. It ranges from 0 to 1 with smaller value indicating weaker association. Features with Cramer's *V* coefficient less than 0.1 were removed. After this procedure, 175 features remained containing 112 PSSM conservation features and 63 secondary structure features, which can be found in Online Supporting Information S3.

### 3.2. The mRMR Result

Two kinds of outcomes were obtained after executing the mRMR program. One was called “MaxRel feature list” that ranked the features according to the relevance to the target; the other was named “mRMR feature list” that ranked the features based on the criteria of maximum relevance and minimum redundancy. In our research, only the “mRMR feature list” was used to select optimal feature subset in the IFS procedure. It was listed in Online Supporting Information S4.

### 3.3. IFS and Optimal Feature Subset

**Figure 1:**
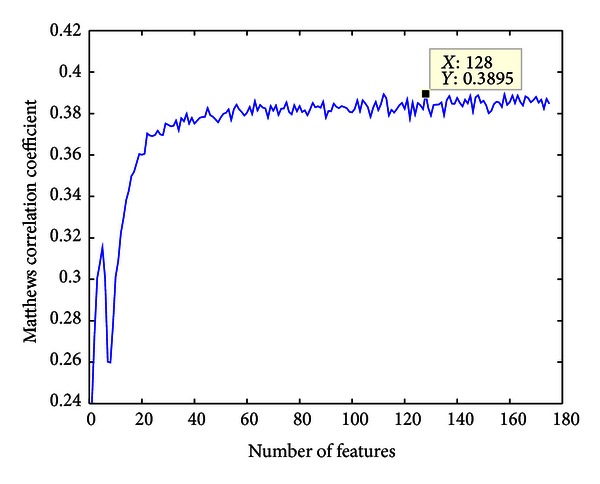
The IFS curve showing the Matthews correlation coefficient (MCC) against the number of features. The details were given in Online Supporting Information S5. With the top 128 features, the MCC on training set by 10-fold cross-validation takes the peak 0.3895.

175 predictors were constructed based on the 175 feature subsets in the IFS procedure. Prediction results of the predictors were listed in Online Supporting Information S5 and the IFS curve was plotted in Figure 1 in which the MCC reached the topmost 0.3895 with 128 features on the training set. Thus, the top 128 features were considered as the optimal feature subset and were used to construct the final predictor. The 128 features were given in Online Supporting Information S6. The MCC of the predictor on independent test set was 0.2791.

### 3.4. Feature Analysis

**Figure 2:**
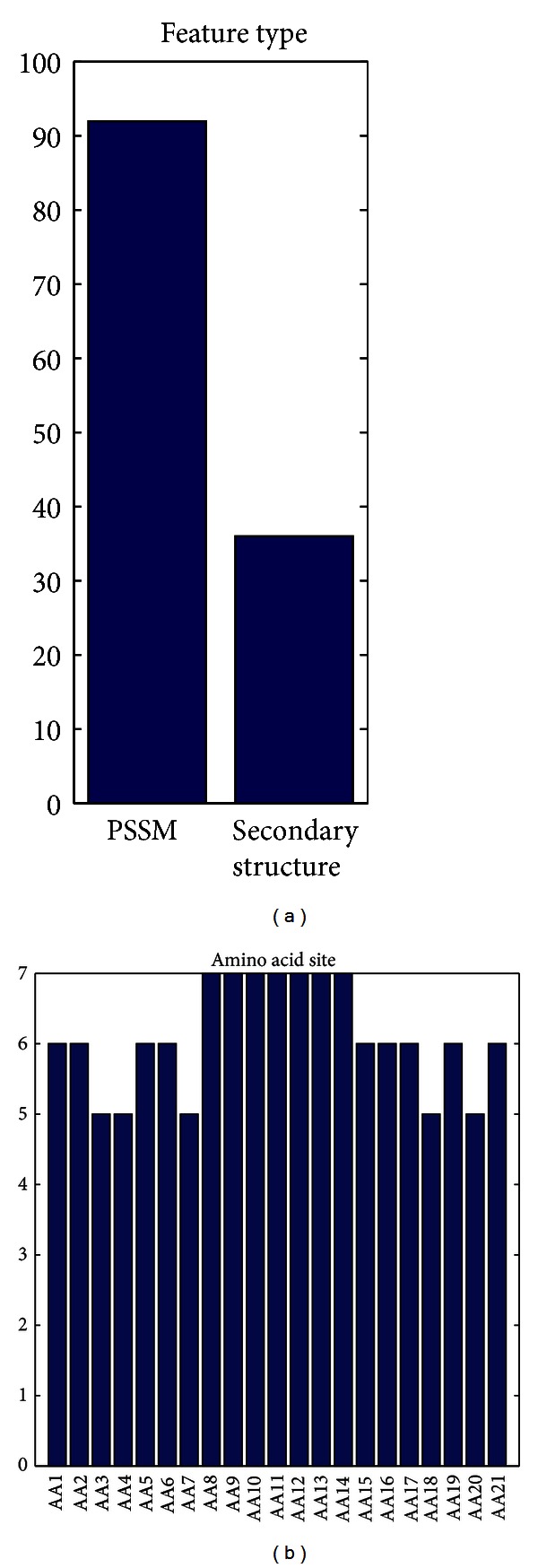
The distribution of feature types and amino acid sites in optimal feature subset. The histograms show the number of each type and each site of features in optimal feature subset. In (a), there are 92 PSSM features and 36 secondary structure features. (b) provides the site distributions of the features in the optimal feature set.

The distribution of the feature types in the final optimal feature set was shown in Figure 2. In the 128 optimal features, 92 were from PSSM conservation scores and 36 from secondary structure features (Figure 2(a)). The two types of features contributed to the prediction. It can be seen from the site-specific distribution of the optimal feature set (Figure 2(b)) that features at sites 8–14 played important roles. In addition, features at sites 1-2, sites 5-6, and sites 15–17, 19, and 21 also had considerable impacts on the prediction of disordered protein.

### 3.5. PSSM Conservation Score Feature Analysis

**Figure 3:**
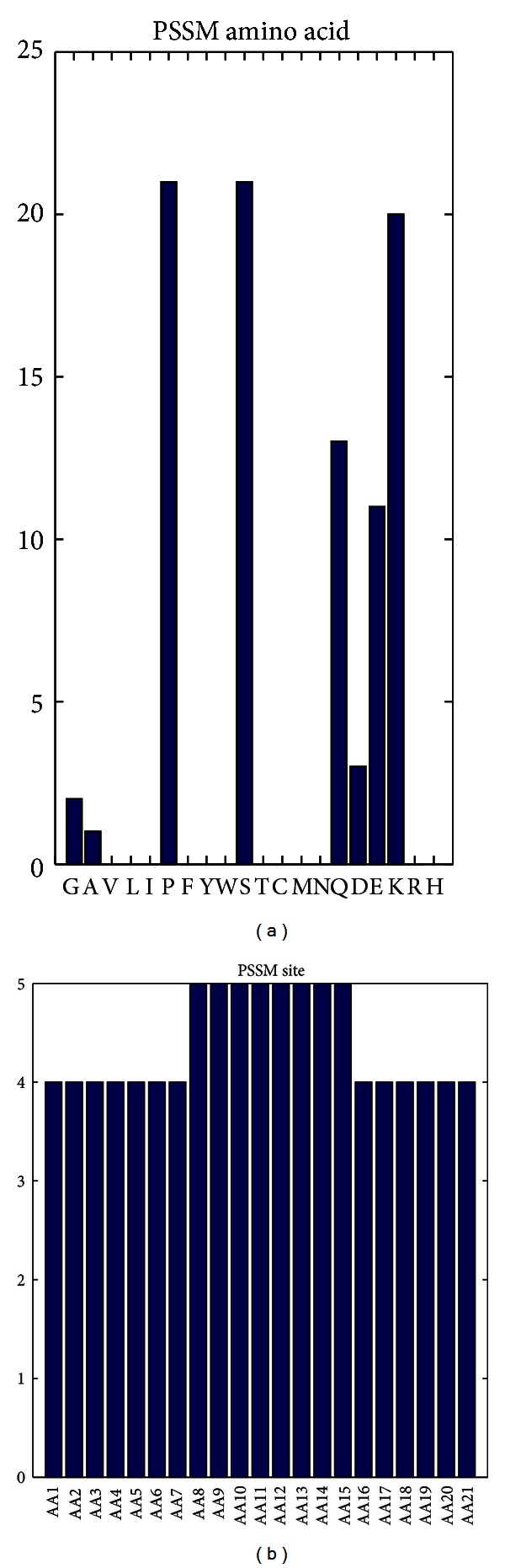
The distribution of amino acid compositions and sites on PSSM conservation feature. The histograms reveal the types and site distributions of PSSM features in the optimal feature set. (a) indicates the effects on prediction of mutations to 20 different amino acids. (b) provides the site distributions of the PSSM features in the optimal feature set.

As mentioned above, among the 128 optimal features, 92 belonged to the PSSM conservation scores, accounting for the most. Mutations to 20 different amino acids could have different impacts in determining the disordered regions. It can be clearly seen from Figure 3(a) that only 8 out of 20 amino acid mutations were affected. In this regard, the amino acid P (Proline) or S (Serine) could impact most, successively followed by K (Lysine), Q (Glutamine), and so forth. Interestingly, it has been reported that Q was overrepresented in protein interaction domains [37]. It was recently reported that the Ure2p prion and other Q/N-rich yeast prion proteins, which were completely disordered, were driven to format amyloid primarily by intermolecular interactions [38]. Meanwhile, as shown in Figure 3(b), for the 21-length peptides, PSSM conservation scores at sites 8–15 played the most important role. Furthermore, 6 out of the top 10 features in the optimal feature list were PSSM conservation features. The first one was the conservation feature against residue K (Lys) at site 6 (index 2, “AA6_PSSM-12-K”). The other 5 were conservation features against residues E, P, and K at sites 7, 1, and 8, respectively (index 5, “AA7_PSSM-7-E”, index 6, and index 7, “AA1_PSSM-15-P” and “AA8_PSSM-12-K”) and conservation features against residue E, D at site 21 and site 15 (index 6 and index 7, “AA21_PSSM-7-E” and “AA15_PSSM-4-D”).

### 3.6. Secondary Structure Feature Analysis

**Figure 4:**
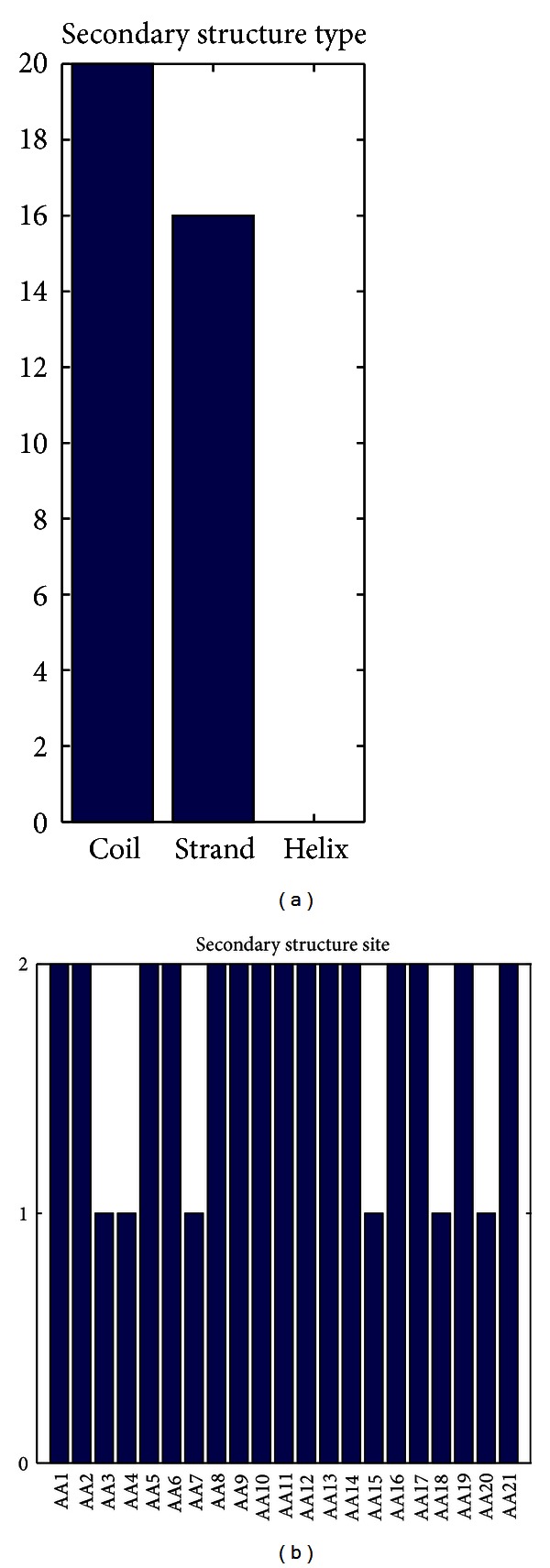
The distribution of secondary structure types and amino acid sites on secondary structure feature. The histograms give the types and site distributions of secondary structure features in the final optimal feature set. (a) indicates the effects on prediction of three different types of secondary structures: coil, strand, and helix. (b) provides the site distributions of the secondary structure features in the optimal feature set.

The feature subtypes and site-specific distributions of the secondary structure features in the optimal feature set were plotted in Figure 4. From Figure 4, it can be seen that features of “coil” and “strand” did affect the disorder (Figure 4(a)). And the “coil” feature was affected the most, followed by the “strand” feature. The secondary structure features at 15 out of 21 sites had relatively more impact than the left 5 (Figure 4(b)). Intrinsically disordered and aggregation-prone domains exist within the very diverse set of human extracellular matrix protein [39]. Recently Evans reported that the aragonite extracellular matrix proteins (AECMPs) had evolved signature molecular traits of intrinsically disordered and aggregation-prone “interactive” sequences that enabled matrix assembly [40]. It was also reported that cyclization of the skeletal DHPR II-III loop affected the secondary structures and the dynamic properties of the helical A/B region as well as the critical C region. These structural effects were correlated with a change in vitro activation profile of the RyR1 and with an interaction with DHPR II-III loop *α*-helical recognition sites in the SPRY2 domain of RyR1 [41]. So it is believed the sequence location and number of intrinsically disordered and secondary motifs may be important for aggregation, protein orientation, and assembly stability and may also play a role in the recognition and interaction between proteins with other specific component(s).

### 3.7. Scan the Entire Protein Sequence to Refine the Disordered Region Prediction

The prediction result of the predictor constructed based on the 128 optimal features on the independent test was shown in Online Supporting Information S7. The third column, *predicted*, was the prediction result where “1” indicated the residue was in ordered region while “2” denoted the residue was in disordered region. It can be seen that many ordered (1) sites were wrong predicted as disordered (2), resulting in short disordered segments (2) being inserted in an ordered segment (1), and vice versa. Therefore, we used a scanning method to refine the prediction results according to the following criteria [42]. (i) Any predicted disordered sites (2) were refined to ordered (1) if there were more than 4 continuous “1s” upstream of the site but less than 4 continuous “2s” downstream of it. (ii) Any predicted ordered sites (1) were changed to disordered (2) if there were more than 4 continuous “2s” upstream of the site but less than 3 continuous “1s” downstream of it. After the refinement procedure, the performance improved much as shown in Table 1. The scanning results can be found in the last column, *scanning*, also can be found in Online Supporting Information S7.

### 3.8. Comparison with the Existing Methods

Our method was compared with three other existing methods, DISOPRED, DISOclust, and OnD-CRF. The DISOPRED server allows users to submit a protein sequence and returns a probability estimate of being disordered of each residue in the sequence [11]. In the prediction results by DISOPRED, disordered residues were marked with asterisks (∗) and ordered residues were marked with dots (·). The prediction results by DISOclust were formulated by a series of “D” and “O,” denoting the residues being in disordered region and ordered region, respectively. The predicting result by Ond-CRF only delivers users the information of disordered regions. As a result, our method outperformed the other three existing methods. As shown in Table 1, the ACC and MCC were improved to a certain extent, at least 4% increase on ACC and almost 0.2% increase on MCC. It is suggested that our method is pretty more effective than other methods on prediction of intrinsically disordered protein region.

### 3.9. Useful Insights for Guiding Experiments or Being Validated by Experiments

About 50% of human proteins were previously predicted to contain at least one larger disordered region, and it was shown that the main reason for the existence of such regions was to harbor binding sites [43]. In this study, the selected features at different sites could provide insights for researchers to find or validate new disordered protein or disordered regions, as can be seen from the following two aspects. (i) PSSM feature: it was found from the results that the PSSM conservation score that mutates to amino acid P or S had the most impact. Besides, mutations to amino acids K, Q, and E also had more impacts than others. However mutations to other 12 amino acids were affected little. For example, phosphorylation of Ser66 in the intrinsically disordered N-terminal region of AtREM1.3 weakened the interaction strength with importin *α* proteins, indicating a regulatory domain in the N-terminal region stabilizing the interactions [44]. (ii) Secondary structure feature: it was found in our optimal feature set that the second structure feature at site 11 had the ranking index 1, implying the most important role to the prediction. Interestingly, it has been reported that disorder regions often correlated domain boundaries where usually harbor some coil structures [45]. Accordingly, other features in the optimal feature set are certainly worth being further investigated by future experiments.

## 4. Conclusion

The plasticity of disordered regions provides interaction capacity. In this study, we investigated important features for predicting disordered protein regions. As a result, the PSSM conservation scores and the second structures are two types of important features, which play key important roles in determining disordered regions. Among these, only 8 amino acids play major roles. The coil and strand structures also affected the prediction. These may provide additional insight into disordered proteins.

## Supplementary Material

Online Supporting Information S1: The training dataset contains 43,903 ordered samples and 43,903 disordered samples of amino acid residues by 21-residue sliding window.
Online Supporting Information S2: The testing dataset contains 54,582 ordered samples and 11,734 disordered samples of amino acid residues by 21-residue sliding window.
Online Supporting Information S3: After calculating the Cramer's V coefficient between features and target variables and removing the features with the Cramer's V coefficient small than 0.1, 175 features remained.
Online Supporting Information S4: The mRMR ranks of the 175 features.
Online Supporting Information S5: The IFS results on training set evaluated by 10-fold cross validation, ACC (Accuracy), MCC (Matthews correlation coefficient), SN (sensitivity), SP (specificity) included.
Online Supporting Information S6: The optimal feature subset includes 128 features.
Online Supporting Information S7: The prediction specific outcome of each residue on testing set. The first 5 columns are the output by WEKA program and the last is the prediction result after scanning the sequence.

## Figures and Tables

**Table 1:** The evaluation of prediction result on independent test set by different methods.

Method	Accuracy (ACC)	Matthews correlation coefficient (MCC)	Sensitivity (SN)	Specificity (SP)
Before scanning	0.7028	0.2791	0.7189	0.6281
After scanning	0.7508	0.3304	0.7806	0.6118
DISOPRED	0.7173	0.3285	0.7239	0.6864
DISOclust	0.6650	0.3105	0.6453	0.7570
OnD-CRF	0.6562	0.3228	0.6265	0.7941
